# Staged external and internal locked plating for open distal tibial fractures: A retrospective study of 16 patients

**DOI:** 10.3109/17453674.2010.487244

**Published:** 2010-05-21

**Authors:** Ching-Hou Ma, Shang-Won Yu, Yuan-Kun Tu, Cheng-Yo Yen, James Jih-Hsi Yeh, Chin-Hsien Wu

**Affiliations:** Department of Orthopedics, E-Da Hospital and I-Shou University, KaohsiungTaiwan

## Abstract

**Background and purpose:**

Based on reported success with staged treatment of distal tibial fractures, we designed a 2-stage protocol including external/internal locked plating. We retrospectively assessed the outcome of open distal tibial fractures treated according to this protocol.

**Patients and methods:**

From March 2006 through July 2008, 16 patients who sustained open distal tibial fractures were treated by a two-stage protocol. The first stage consisted of low-profile, locked plates for temporary external fixation after debridement and anatomic reduction, followed by soft tissue reconstruction. The second stage consisted of locked plates for definitive internal fixation, using minimally invasive percutaneous osteosynthesis. All fractures were followed for median 2 (1–3) years.

**Results:**

The reduction was classified as being good in 15 patients and fair in 1 patient. All fractures united at a median of 6 (6–12) months. At the latest follow-up, 7 patients had excellent and 9 had good Iowa ankle scores; ankle motion ranged from a median of 10 (5–20) degrees of dorsiflexion to 40 (20–60) degrees of plantar flexion.

**Interpretation:**

We believe that the 2-stage external/internal locked plating technique is an effective procedure for treatment of open distal tibial fractures in patients who need a long period of external fixation. We achieved good reduction with immediate ankle-sparing stable fixation. Soft tissue reconstruction and subsequent definitive fixation led to union of all fractures with good function.

## Introduction

In open distal tibial fracture, several authors have demonstrated the benefits of bridging external fixation followed by definitive internal fixation once the soft tissue has healed sufficiently (Pugh et al. 1999, Sirkin et al. 1999, Haidukewych 2002).

Low-profile external fixation without ankle spanning is a helpful adjunct in these fractures, as part of a staged reconstruction (Apivatthakakul and Sananpanich 2007). A locking plate, such as the less invasive stabilization system (LISS) plate, is also a good candidate for external plate fixation (Kloen 2009). Based on the success with staged management of distal tibial fractures (Apivatthakakul and Sananpanich 2007), we designed a 2-stage protocol for open type II and type III distal tibia fractures (Gustilo and Anderson 1976).

We retrospectively evaluated the outcome of using the locked plate as a temporary external fixator for type II and type III open, distal tibial fractures with regard to soft tissue reconstruction, followed by definitive minimally invasive percutaneous fixation.

## Patients and methods

From March 2006 through July 2008, we treated 16 consecutive patients (9 women) with open distal tibial fractures using our staged protocol (Table 1). These patients could not be treated with primary wound closure (n = 5) or had progressive wound necrosis in the first days after primary wound closure (n = 11). All fractures needed long-term external fixation because of severe soft tissue injury initially or progressive soft tissue necrosis. Patients with infections or nonunion following surgery in other institutes and fractures in children were not included. The median age at time of surgery was 56 (26–77) years. The fractures were caused by falls from > 2 meters in height (n = 2), machinery-crushing injury (n = 1), or traffic accidents (n = 13). 5 cases had other organ injuries. 12 were Gustilo and Anderson type III (7 type IIIA, 4 type IIIB, and 1 type IIIC) and 4 were type II (Gustilo and Anderson 1976, Gustilo et al. 1984). According to the AO/OTA classification (Müller et al. 1990), there were 7 type C fractures (2 type C3 fractures and 5 type C2), and 9 type A fractures (8 type A3 fractures and 1 type A2).

**Table 1.** Patient demographics

Case	Sex/Age	Mechanism ^**a**^	Gustilo grade	AO/OTA classification	Comorbidity ^**b**^	Other fractures	Associated injury
1	M/68	TA	IIIB	43-C2	Smoker	Femur	Epidural hemorrhage
2	F/62	TA	IIIA	43-C2	–	–	–
3	F/77	TA	II	43-C2	HTN, DM	–	–
4	M/30	Fall	IIIA	43-C3	Smoker	Radius	–
5	M/39	TA	IIIB	43-C2	–	–	–
6	M/52	TA	IIIA	43-C3	Smoker	–	Subarachnoid hemorrhage
7	F/50	TA	II	43-C2	–	Toe	_
8	F/72	TA	IIIB	43-A3	–	–	_
9	F/60	TA	IIIB	43-A3	–	Femurs, tibia	_
10	F/67	TA	IIIA	43-A3	–	Femur	Intracranial hemorrhage
11	M/35	Fall	II	43-A3	–	Lumbar spine, calcaneus	–
12	M/42	Crushing	IIIA	43-A2	–	–	–
13	F/26	TA	II	43-A3	–	Pelvis	Blunt abdominal trauma
14	M/38	TA	IIIC	43-A3	–	Radius, humerus, femur	Hemopneumothorax
15	F/62	TA	IIIA	43-A3	HTN	–	–
16	F/69	TA	IIIA	43-A3	DM	Metatarsal bone	–

^**a**^TA: traffic accident.

^**b**^HTN: hypertension; DM: diabetes mellitus.

### Protocol (Figure 1)

#### Stage 1

After immediate irrigation and debridement, in unstable patients the fracture was stabilized with standard external fixators. Reduction was done through the wound, with a short extended incision if needed for access. Limited intraarticular fragment fixation was performed using 3.5-mm screws. If necessary, fibular fixation was performed percutaneously with pins, cannulated screws, or plate. Then a non-spanning external fixator was applied, either a LISS-distal femur plate (Synthes, Paoli, PA) or an external locked plate (Kaohsiung, Taiwan). The locked plate was placed on the opposite side of the wound, so as not to interfere with the soft tissue reconstruction to follow. Bicortical fixation was used (Figure 2C).

**Figure 1.**
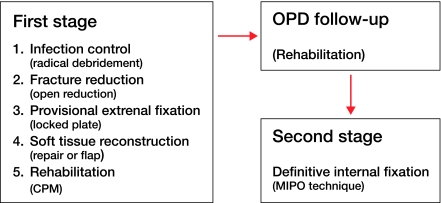
Flow diagram of treatment protocol.

**Figure 2.**
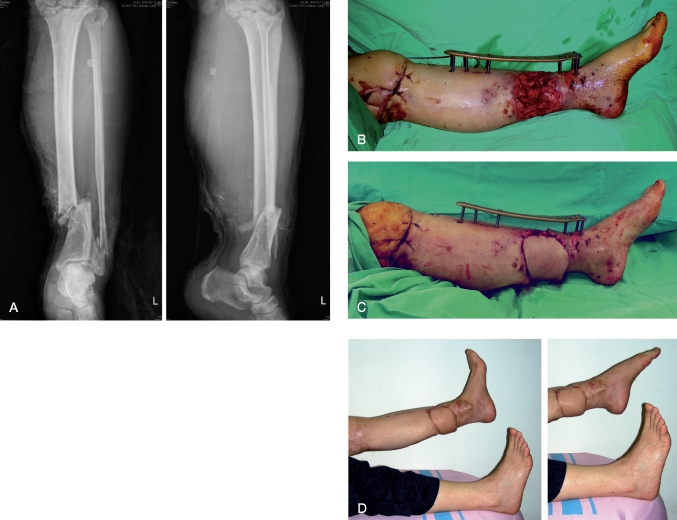
A 60-year-old woman (case 9) sustained a Gustilo type IIIB, AO/OTA 43-A3 fracture (A). The fracture was stabilized with a LISS-DF plate used as an external fixator (B), and then the soft tissue defect was treated with an anterolateral thigh free flap (C). The functional outcome was excellent at the 6-month follow-up visit (D).

**Figure 3.**
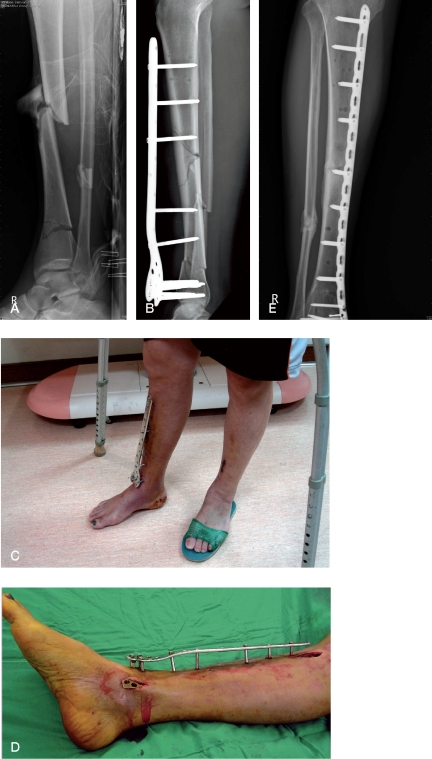
A 42-year-old man (case 12) sustained a Gustilo type IIIA, AO/OTA 43-A2 fracture (A). The fracture was stabilized with a locked plate as an external fixator (B). The low-profile locked plate provided enough stability with little limitation of dressing, or walking (C). The definitive internal fixation was performed using a MIPO technique with a metaphyseal locked plate (D). There was union without malalignment at the 1-year follow-up visit (E).

Although no specific protocol for soft tissue coverage was used, all patients were treated within 7 days of admission. 7 patients had only wound repair, 6 patients had reverse sural artery fasciocutaneous flaps, 2 patients had latissimus dorsi free flaps, and 1 patient had an anterolateral thigh free flap (Figures 2 and 3).

#### Stage 2

All patients were admitted for definitive fixation using minimally invasive percutaneous osteosynthesis with distal tibial/metaphyseal plates (Synthes, Paoli, PA) when the soft tissue had healed after median 7 (4–14) weeks (Figure 3).

Clinical and radiographic follow-up examinations were performed at 3 months, 6 months, 1 year, and at 2 years in 10 patients, and at 3 years in 1 patient.

### Clinical assessment

The Iowa ankle questionnaire (Merchant and Dietz 1989) was answered at 1 year and at the final follow-up. This clinical rating system has separate scores for pain and function. A score of 85–100 represents an excellent result, 70–84 a good result, 60–69 a fair result, and less than 60 a poor result.

### Radiographic assessment

Reduction of the articular surface was classified according to the modified Burwell and Charnley system (Marsh et al. 1995). In the extraarticular fractures, displacement was classified as excellent (< 2 mm), good (< 5 mm), fair (< 10 mm) and poor (≥ 10 mm). The Johnson angle was measured in both planes (Möller 1999). More than 5 degrees deviation was defined as malunion. We defined union as more than 50% visible bridging callus across the fracture on conventional radiographs (Panjabi et al. 1985). At final follow-up, any ankle arthrosis was radiographically graded (Marsh et al. 1995).

## Results (Table 2)

The median hospital stay was 21 (14–49) days; it was 19 days for patients who had orthopedic injuries only and 30 days for those who had other injuries.

### Clinical outcome

At final follow-up, the median Iowa ankle score was 83 (73–89). 7 patients had excellent scores and 9 had good scores. Mean ankle motion ranged from 10 (5–20) degrees of dorsiflexion to 40 (20–60) degrees of plantar flexion.

### Radiographic outcome

15 patients had good articular or fracture reduction. 1 patient had fair articular reduction. At the final follow-up, 1 patient had 5 degrees of angulation. 11 patients had grade 1 ankle arthrosis and 2 patients had grade 2 ankle arthrosis. All fractures had united at a median of 6 (6–12) months.

### Complications

Postoperative complications included minor screw track infections in 2 patients, which resolved with oral antibiotic treatment and care of screw sites. There was no loosening of external fixators due to screw track infection. 2 patients had superficial infections that resolved with parenteral antibiotic treatment. No deep infections or flap failures occurred.

## Discussion

Two main external fixation devices have been developed for the treatment of distal tibial fractures. One is the ankle-sparing system, a hybrid of the unilateral frame and the Ilizarov system, and the other is the ankle-spanning system, a unilateral frame with pins in the medial tibial shaft and the talus and calcaneus.

An Ilizarov-type device maintains the reduction of fractures, eliminates the need for implanted hardware, and provides a stable platform for soft tissue reconstruction (Ristinemi et al. 2007, Bozkurt et al. 2008). The main disadvantage of the Ilizarov method is the long treatment time.

Patients treated with spanning frames more often have loss of reduction than patients treated with sparing frames (Pugh et al. 1999, Papadokostakis et al. 2008). However, the open distal tibial fracture is often associated with multiple organ injuries, which prolong the time before definitive internal fixation can be done. Thus, the spanning external fixators have to stay in place for a long time, which may result in ankle stiffness (Rommens et al. 1989, Koulouvaris et al. 2007).

Apivatthakakul and Sananpanich (2007) and Kloen (2009) described locked plates as external fixators for the treatment of infected nonunion and open fractures. This technique was well tolerated by patients. The authors suggested that it could be a useful adjunct in the staged treatment of complex reconstructive cases. The design of the LISS plate is suitable to stabilize short metaphyseal segments because many metaphyseal screw holes can be chosen, depending on the fracture pattern.

We achieved a high rate of anatomic reduction of the ankle joint and fracture site by reduction through the open wound or a short extended incision. Since the locked plate provided enough stability and did not cross the ankle joint, rehabilitation could be started early. The total duration of hospitalization was relatively short. Replacement of temporary external fixators with definitive internal fixators was fast and easy, because reduction was already done. The patients stayed in the hospital for around 1 week.

The use of locked plates as temporary external fixators is not a generally acknowledged technique, and there is little experience with it in the literature. We found a high rate of union with a low complication rate when using staged external and internal locked plating for open distal tibial fractures.

**Table 2.** Clinical and radiographic results

A	B	C	D	E	F	G	H	I	J	K
1	24	LD	Good	86	48 + 8	Screw track infection	12	90/85	Excellent	10–40
2	24	RS	Good	36	13 + 7	–	12	89/78	Excellent	15–50
3	24	Repair	Good	27	9 + 5	Superficial infection	6	91/80	Excellent	10–30
4	12	RS	Good	60	13 + 6	–	6	91/81	Good	5–20
5	24	RS	Good	97	8 + 7	–	9	89/80	Good	15–60
6	12	RS	Fair	62	44 + 5	Superficial infection	6	90/78	Good	5–30
7	24	Repair	Good	26	8 + 6	–	6	88/75	Excellent	15–50
8	24	RS	Good	28	10 + 9	–	6	87/77	Excellent	10–40
9	36	ALT	Good	83	36 + 7	–	12	94/77	Excellent	5–30
10	24	Repair	Good	32	22 + 8	–	6	90/80	Good	10–40
11	24	Repair	Good	27	21 + 8	Screw track infection	6	91/79	Good	20–50
12	12	Repair	Good	47	9 + 6	–	12	95/81	Good	10–30
13	12	Repair	Good	31	13 + 9	–	6	90/80	Good	10–40
14	24	LD	Good	54	26 + 7	–	6	93/80	Excellent	10–50
15	24	RS	Good	97	11 + 9	–	6	87/84	Good	10–40
16	12	Repair	Good	51	27 + 11	–	12	90/75	Good	10–20

A Case B Follow-up (months) C Soft tissue reconstruction LD: latissimus dorsi free flap RS: reverse sural artery fasciocutaneous flap ALT: anterolateral thigh free flap D Articular reduction E Temporary fixation (days) F Duration of hospitalization: first + second stage (days) G Complications H Time to union (months) I Axis J Iowa ankle score K Range of motion (degrees)

## References

[CIT0001] Apivatthakakul T, Sananpanich K (2007). The locking compression plate as an external fixator for bone transport in the treatment of a large distal tibial defect: case report. Injury.

[CIT0002] Bozkurt M, Ocquder DA, Ugurlu M (2008). Tibial pilon fractures reapir using Ilizarov external fixation, capsuloligamentotaxis, and early rehabilitation of the ankle. J Foot Ankle Surg.

[CIT0003] Gustilo RB, Anderson JT (1976). Prevention of infection in the treatment of one thousand and twenty-five open fractures of long bones: retrospective and prospective analyses. J Bone Joint Surg (Am).

[CIT0004] Gustilo RB, Mendoza RM, Williams DN (1984). The management of type III (severe) open fractures: a new classification of type III open fractures. J Trauma.

[CIT0005] Haidukewych GJ (2002). Temporary external fixation for the management of complex intra-and periarticular fractures of the low extremity. J Orthop Trauma.

[CIT0006] Kloen P (2009). Supercutaneous plating: Using a locking compression plate as an external fixator. J Orthop Trauma.

[CIT0007] Koulouvaris P, Stafylas K, Mitsionis G (2007). Long-term results of various therapy concepts in severe pilon fractures. Arch Orthop Trauma Surg.

[CIT0008] Marsh JL, Bonar S, Nepola JV (1995). Use of an articulated external fixator for fractures of the tibial plafond. J Bone Joint Surg (Am).

[CIT0009] Merchant TC, Dietz FR (1989). Long-term follow-up after fractures of the tibial and fibular shaft. J Bone Joint Surg (Am).

[CIT0010] Möller T, Möller T (1999). Unfere Extremitäten. Röntgennormalbefunde.

[CIT0011] Müller ME, Nazarian S, Koch P (1990). The comprehensive classification of fractures of long bones.

[CIT0012] Panjabi MM, Walter SD, Karuda M (1985). Correlations of radiographic analysis of healing fractures with strength: a statistical analysis of experimental osteotomies. J Orthop Res.

[CIT0013] Papadokostakis G, Kontakis G, Giannoudis P (2008). External fixation devices in the treatment of fractures of the tibial plafond: a systemic review of the literature. J Bone Joint Surg (Br).

[CIT0014] Pugh KJ, Wolinsky PR, McAndrew MP (1999). Tibial plafond fractures: a comparison of treatment methods. J Trauma.

[CIT0015] Ristiniemi J, Flinkkilä T, Hyvönen P (2007). Two-ring hybrid external fixation of distal tibial fractures: A review of 47 cases. J Trauma.

[CIT0016] Rommens P, Gielen J, Broos P (1989). Intrinsic problems with the external fixation device of Hoffmann-Vidal-Adrey: a critical evaluation of 117 patients with complex tibial shaft fractures. J Trauma.

[CIT0017] Sirkin M, Sanders R, Dipasquale T (1999). A staged protocol for soft tissue management in the treatment of complex pilon fractures. J Orthop Trauma.

